# Associations of Dietary and Circulating Choline and Glycine With Bone Mineral Density: UK Biobank Analyses With Mendelian Randomization

**DOI:** 10.1155/ije/2237464

**Published:** 2026-04-10

**Authors:** Siyu Chen, Rui Lu, Yazhong Zhang, Yusheng Li, Xueqin Cao, Huilin Yang, Jun Lin

**Affiliations:** ^1^ Department of Endocrinology and Metabolism, The Fourth Affiliated Hospital of Soochow University (Suzhou Dushu Lake Hospital), Medical Center of Soochow University, Suzhou, Jiangsu, China; ^2^ Department of Radiology, Children’s Hospital of Nanjing Medical University, Nanjing, Jiangsu, China, njmu.edu.cn; ^3^ Department of Orthopaedics, The Second Affiliated Hospital of Xuzhou Medical University, Xuzhou, Jiangsu, China, xzmc.edu.cn; ^4^ Department of Orthopaedics, Xiangya Hospital, National Clinical Research Center for Geriatric Disorders, Central South University, Changsha, Hunan, China, csu.edu.cn; ^5^ Department of Orthopaedics, The First Affiliated Hospital of Soochow University, Soochow University, Suzhou, Jiangsu, China, scu.edu.tw; ^6^ Department of Orthopaedics, The Fourth Affiliated Hospital of Soochow University (Suzhou Dushu Lake Hospital), Medical Center of Soochow University, Suzhou, Jiangsu, China

**Keywords:** causal inference, nutrient metabolites, observational study, osteoporosis, quantitative ultrasound

## Abstract

**Background:**

Osteoporosis, characterized by reduced bone mass and compromised bone microarchitecture, significantly impacts public health, particularly among the elderly population. Although choline and its related metabolites are believed to influence bone health, their exact role and the effects of these metabolites on bone mineral density (BMD) remain unclear.

**Methods:**

We analyzed data from 18,769 UK Biobank (UKB) participants, excluding those with missing data or specific health conditions. All observational associations were assessed cross‐sectionally at baseline. Choline intake was assessed using a self‐administered 24‐h dietary assessment, while circulating choline and related metabolites were quantified by nuclear magnetic resonance spectroscopy. BMD was measured using ultrasound densitometry. Linear regression models were used to assess the associations, adjusting for various covariates.

**Results:**

Overall, circulating choline levels demonstrated a borderline significant association with BMD (*β* = −0.0056, *p* = 0.052), and glycine levels showed a significantly negative association (*β* = −0.0486, *p* = 0.004). Subgroup analyses revealed that the association between circulating choline and BMD varied by gender, menopausal status, and renal function. Notably, higher choline levels were related to lower BMD in men (*β* = −0.0097, *p* = 0.032) and those with impaired kidney function (*β* = −0.01, *p* = 0.034). While dietary choline intake did not show an overall significant relationship with BMD (*β* = −0.0128, *p* = 0.218), a negative association was observed in individuals with renal impairment (*β* = −0.0385, *p* = 0.027). Furthermore, Mendelian randomization analyses suggested a potential causal association between circulating choline levels and BMD in men (*β* = −0.079, 95% CI: −0.117 to −0.040, *p* < 0.001) and between glycine levels and BMD in women (*β* = −0.022, 95% CI: −0.037 to −0.007, *p* = 0.005).

**Conclusions:**

Choline and glycine metabolism may play a role in bone metabolism, with choline showing particular effects in men and individuals with impaired renal function. Further research is necessary to better understand the underlying mechanisms and implications of these associations for osteoporosis risk in different populations.

## 1. Background

Osteoporosis, characterized by reduced bone mass and damage to bone microarchitecture, poses a significant health burden, with an estimated 40%–50% of women and 13%–22% of men experiencing fragility fractures in their lifetime [1, 2]. These fractures are associated with a prolonged healing process and recovery time, greatly impairing patients’ quality of life and, in severe cases, leading to fatal outcomes [3]. The aging population further exacerbates the urgency to address the pressing public health problem of osteoporosis. However, current treatment options are limited and have not achieved optimal results [4]. Hence, it is essential to prioritize research and development endeavors toward the development of effective and feasible preventative measures alongside innovative treatment options for osteoporosis.

The influence of nutrition on the development and progression of osteoporosis has gained attention, particularly in relation to certain essential nutrients such as calcium, vitamin D, and protein [5, 6]. Among these nutrients, choline—found in dietary sources like eggs, red meat, and dairy products, as well as synthesized within the body—plays a crucial role in human metabolism [7]. Choline is involved in numerous biological processes, including the formation of cell structure and synthesis of neurotransmitters, and its deficiency has been associated with a range of health concerns, including liver disease, neurological disorders, and muscle damage [7–9]. However, recent studies have underscored the complex role of choline, highlighting that high choline levels might be linked with unfavorable cardiometabolic risk profiles and an increased probability of cardiovascular disease, cancer risk, and even mortality [10–12]. Notably, the potential link between choline metabolites and bone metabolism has been scarcely explored. Limited studies conducted among Western populations have reported positive associations between dietary and circulating choline levels and bone mineral density (BMD) [13–15]. Nonetheless, certain choline derivatives, such as the gut bacterial product trimethylamine N‐oxide (TMAO), have demonstrated a negative relationship with BMD in both population and animal studies [16–18]. Meanwhile, circulating TMAO levels are strongly influenced by renal clearance, and TMAO accumulation may be related to impaired kidney function [19]. These observations suggest that associations between choline‐related exposures and bone outcomes may be heterogeneous across exposure levels and population subgroups.

Therefore, we propose a dual hypothesis: Given choline’s essential metabolic roles, within typical intake/physiological ranges, choline‐related exposures may be beneficial or show no significant association with BMD; however, at higher circulating levels or in susceptible subgroups, the association may become unfavorable—for example, among individuals with impaired renal function. Further research is needed to comprehensively understand the effects of choline intake and its metabolites on skeletal health.

Our study aims to investigate the relationship between dietary choline, circulating levels of choline, phosphatidylcholine, glycine, and BMD in a large‐scale study among the UK population aged between 40 and 70 years.

## 2. Methods

### 2.1. Study Population

The study population included participants from the UK Biobank (UKB). Between 2006 and 2010, over 500,000 individuals aged 37–73 years were recruited. For the present study, participants without data on circulating choline (*n* = 383,501), dietary choline (*n* = 290,718), BMD (*n* = 222,780), or menopausal status (*n* = 42,784) were excluded. Additionally, considering that certain endocrine disorders may affect BMD and introduce residual confounding that may not be fully addressed by covariate adjustment, participants with preexisting conditions such as diabetes [20] at baseline (*n* = 26,166), hyperparathyroidism [21] (*n* = 641), hyperthyroidism [22] (*n* = 5,392), or Cushing syndrome [23] (*n* = 125) were excluded. Disease identification was based on Read codes from primary care data, ICD‐9 and ICD‐10 codes from hospitalization records, and self‐reported disease codes. Participants with extreme energy intakes (greater than 20,000 kJ for men and 18,000 kJ for women) were also excluded (*n* = 581). Finally, 18,769 participants were included for analysis.

### 2.2. Assessment of Dietary Intake

Dietary data were collected using the Oxford WebQ, a web‐based 24‐h dietary recall tool, with initial assessment conducted at the assessment center (2009–2010), followed by up to four additional online assessments (2011–2012) for participants with valid email addresses. The Oxford WebQ allowed participants to report their intake by selecting the number of portions consumed from a list of 206 commonly consumed foods and 32 beverages in the United Kingdom. This tool has been validated against biomarkers and compared to interviewer‐administered 24‐h recalls, demonstrating acceptable reproducibility across multiple assessments [24]. Mean dietary values were calculated based on the available data. Nutrient intakes, including energy, protein, potassium, calcium, and vitamin D, were estimated using built‐in algorithms and food composition data from The Composition of Foods 6th edition [25]. To estimate dietary choline intake, we utilized a comprehensive food grouping system developed for the database and supplemented our data with the USDA Database for the Choline Content of Common Foods [26]. Further details are provided in Supporting Methods.

### 2.3. Assessment of Circulating Choline and Its Related Metabolites

Circulating choline, phosphatidylcholine, and glycine were measured in a random subset of plasma samples collected between 2007 and 2010. These measurements were performed using a high‐throughput nuclear magnetic resonance (NMR)–based metabolic biomarker profiling platform developed by Nightingale Health Ltd.

### 2.4. Measurements of BMD

BMD of the heel was assessed using ultrasound densitometry (QUS) with the Sahara bone sonometer. Trained staff conducted the measurements using the following formula: BMD = 0.002592 × (BUA + SOS) − 3.687 g/cm^2^, where BUA refers to broadband ultrasound attenuation (dH/MHz) and SOS denotes the speed of sound (m/s). Measurements were performed on both the left and right calcaneus, and the average of these values was used for analysis.

### 2.5. Covariates

Covariates are described in Supporting Methods.

### 2.6. Statistical Analysis

The observational analyses were cross‐sectional, relating baseline dietary/circulating biomarkers to contemporaneous heel ultrasound‐derived BMD. Continuous variables were presented as means with standard deviations, while categorical variables were expressed as frequencies with percentages. To determine the *p* value for trend, Spearman’s correlation was applied to continuous variables, while the Mantel–Haenszel test was used for categorical variables. Pearson correlation and Pearson partial correlation analyses (adjusted for age and sex) were performed to evaluate the relationships between dietary choline intake and circulating choline levels.

Linear regression models were used to examine the associations between dietary intake of choline, circulating choline levels, and glycine levels with BMD. The analyses were conducted using the following three models:

• Model 1: Adjusted for age, sex, menopausal status, race, annual household income, qualification, fasting time, smoking status, alcohol consumption, and physical activity.
• Model 2: Adjusted for all variables in Model 1, plus BMI, regular use of vitamin D and/or multivitamin supplements, regular use of calcium supplements, and dietary intake of energy, protein, potassium, calcium, and vitamin D. For women, hormone replacement therapy (HRT) use was also included.
• Model 3: Adjusted for all variables in Model 2, plus estimated glomerular filtration rate (eGFR) and plasma vitamin D levels.

Subgroup analyses were performed to assess associations stratified by sex, menopausal status, liver function (ALT/AST ≤ 40 U/L vs. ALT/AST > 40 U/L), and renal function (eGFR ≥ 90 mL/min/1.73 m^2^ vs. eGFR < 90 mL/min/1.73 m^2^). To investigate potential multiplicative effect modification by these stratification variables, likelihood ratio tests were used to compare models before and after introducing interaction terms between the exposure and each stratification variable in Model 3. No imputation was performed for missing covariates. Adjusted significance thresholds were two‐sided *p* < 0.05. All analyses were conducted by using the R software, Version 4.0.4.

### 2.7. Mendelian Randomization (MR)

Results from conventional observational studies may be affected by confounding factors or reverse causation, which can be mitigated to some extent through MR analysis. Thus, MR was employed to evaluate potential causal relationships between choline metabolites and BMD, based on the findings obtained through the aforementioned approach. MR is an epidemiological study design in which genetic variations associated with exposure factors are used as instrumental variables to assess potential causality and outcomes [27]. The alleles of a given single‐nucleotide polymorphism (SNP) are randomly assigned to egg/sperm cells during human gametogenesis, making genetic variants like SNPs suitable instrumental variables. Consequently, inherited variants are independent of potentially confounding environmental exposures [28–30]. A valid genetic instrumental variable fulfills three core assumptions [31]: (1) It must be reproducibly and strongly associated with the exposure; (2) it cannot be associated with confounders; and (3) it is only associated with the outcome through the exposure. We selected genetic variants strongly associated with the exposures (*p* < 5 × 10^−8^) as candidate genetic proxies for these metabolites. We further pruned genetic variants in linkage disequilibrium (*r* ^2^ < 0.001) within a 10000‐kb region. After harmonizing exposure and outcome GWAS data, PhenoScanner (http://www.phenoscanner.medschl.cam.ac.uk/) was used to find and remove SNPs associated with BMD and BMD‐associated traits (e.g., BMI).

GWAS data of choline metabolites including total choline, phosphatidylcholine, and glycine were obtained from UKB (*n* = 114,999). BMD data used in this study were obtained from Neale Lab’s analysis of the UKB dataset, comprising a sample size of 95,344 men and 111,152 women.

“TwoSampleMR” package was applied within R software for Mendelian analysis. The MR analysis used inverse‐variance weighted to obtain *β* values and 95% confidence intervals (CIs). Heterogeneity was assessed using Cochrane’s Q value.

### 2.8. Sensitivity Analysis

To ensure robustness beyond the primary analyses, we conducted two sensitivity analyses. First, we assessed extreme values for circulating choline, phosphatidylcholine, glycine, and heel BMD using Tukey’s rule (1.5 times the interquartile range). Second, in the multivariable model, we performed imputations using the mean to augment the analysis.

## 3. Results

In the study, 18,769 participants were included, with ages ranging from 40 to 70 years. Of these, 9,126 (48.6%) were men, and 17,340 (92.6%) were of the White race. Table 1 presents participants’ characteristics categorized by circulating choline quantiles. Participants with higher circulating choline quantiles were observed to have a higher proportion of women and tended to be older. They were also more likely to engage in physical activity and current drinking. Additionally, they exhibited elevated levels of glycine and were more likely to use vitamin D supplements, calcium supplements, and HRT. On the other hand, participants with higher circulating choline quantiles had lower levels of BMI, serum vitamin D, and dietary intake of energy, protein, and calcium. However, no significant difference was found in the eGFR or dietary intake of choline across different circulating choline quantiles. The characteristics of participants categorized by dietary choline quartiles are shown in Supporting Table 1.

**TABLE 1:** Baseline characteristics of participants according to quantiles of circulating choline levels.

	Total	Circulating choline level				
		Q1	Q2	Q3	Q4	*p* for trend
*N*	18769	4693	4692	4693	4691	
Age, years	55.7 (7.9)	54.7 (8.5)	55.1 (8.0)	55.9 (7.7)	56.9 (7.1)	< 0.001
White race	17340 (92.6%)	4308 (92.1%)	4376 (93.5%)	4339 (92.6%)	4317 (92.3%)	0.846
Men	9126 (48.6%)	3220 (68.6%)	2633 (56.1%)	1963 (41.8%)	1310 (27.9%)	< 0.001
Menopausal status						< 0.001
Premenopausal	3201 (33.2%)	812 (55.1%)	862 (41.9%)	828 (30.3%)	699 (20.7%)	
Postmenopausal	6442 (66.8%)	661 (44.9%)	1197 (58.1%)	1902 (69.7%)	2682 (79.3%)	
Average income per year, £						0.642
< 18000	2236 (11.9%)	583 (12.4%)	554 (11.8%)	572 (12.2%)	527 (11.2%)	
18000–30999	4010 (21.4%)	971 (20.7%)	942 (20.1%)	989 (21.1%)	1108 (23.6%)	
31000–51999	4973 (26.5%)	1253 (26.7%)	1285 (27.4%)	1199 (25.5%)	1236 (26.3%)	
52000–99999	4446 (23.7%)	1130 (24.1%)	1167 (24.9%)	1139 (24.3%)	1010 (21.5%)	
≥ 100000	1343 (7.2%)	345 (7.4%)	345 (7.4%)	345 (7.4%)	308 (6.6%)	
Qualifications						0.001
Higher degree	11524 (61.6%)	2820 (60.3%)	2910 (62.2%)	2859 (61.1%)	2935 (62.7%)	
Any school degree	5036 (26.9%)	1240 (26.5%)	1224 (26.2%)	1294 (27.6%)	1278 (27.3%)	
Vocational qualifications	830 (4.4%)	256 (5.5%)	213 (4.6%)	190 (4.1%)	171 (3.7%)	
Other	1329 (7.1%)	361 (7.7%)	332 (7.1%)	337 (7.2%)	299 (6.4%)	
Smoking status						0.816
Never	10747 (57.4%)	2696 (57.6%)	2711 (57.9%)	2706 (57.7%)	2634 (56.3%)	
Previous	6685 (35.7%)	1630 (34.8%)	1630 (34.8%)	1656 (35.3%)	1769 (37.8%)	
Current	1293 (6.9%)	352 (7.5%)	340 (7.3%)	324 (6.9%)	277 (5.9%)	
Drinking status						< 0.001
Never	468 (2.5%)	145 (3.1%)	107 (2.3%)	125 (2.7%)	91 (1.9%)	
Previous	490 (2.6%)	187 (4.0%)	132 (2.8%)	86 (1.8%)	85 (1.8%)	
Current	17803 (94.9%)	4357 (92.9%)	4451 (94.9%)	4480 (95.5%)	4515 (96.2%)	
Physical activity, MET‐min/week						< 0.001
< 600	2967 (15.8%)	817 (17.4%)	767 (16.3%)	691 (14.7%)	692 (14.8%)	
600–2999	8898 (47.4%)	2260 (48.2%)	2229 (47.5%)	2217 (47.2%)	2192 (46.7%)	
≥ 3000	4270 (22.8%)	1013 (21.6%)	1080 (23.0%)	1093 (23.3%)	1084 (23.1%)	
BMI (kg/m^2^)	26.6 (4.4)	27.1 (4.5)	26.6 (4.3)	26.5 (4.4)	26.2 (4.2)	< 0.001
Regular vitamin D supplement use	4630 (24.7%)	1010 (21.6%)	1147 (24.5%)	1189 (25.4%)	1284 (27.4%)	< 0.001
Vitamin D (nmol/L)	48.3 (20.3)	49.7 (21.2)	48.5 (20.8)	47.6 (19.9)	47.3 (19.3)	< 0.001
Regular calcium supplement use	1302 (6.9%)	206 (4.4%)	265 (5.7%)	352 (7.5%)	479 (10.2%)	< 0.001
Hormone replacement therapy	3096 (32.1%)	319 (21.7%)	587 (28.5%)	912 (33.4%)	1278 (37.8%)	< 0.001
Daily dietary intake						
Energy (kJ/day)	8713.0 (2353.2)	8949.9 (2438.6)	8839.9 (2394.3)	8616.9 (2324.5)	8445.0 (2217.3)	< 0.001
Protein (g/day)	81.3 (23.5)	83.5 (24.6)	82.0 (23.8)	80.4 (23.0)	79.5 (22.5)	< 0.001
Potassium (mg/day)	3690.6 (1025.1)	3704.2 (1046.8)	3697.7 (1037.1)	3685.0 (1017.7)	3675.7 (998.2)	0.142
Calcium (mg/day)	984.3 (327.6)	1012.0 (338.2)	998.6 (339.1)	973.0 (318.6)	953.7 (310.5)	< 0.001
Vitamin D (μg/day)	3.7 (2.9)	3.8 (3.0)	3.7 (2.8)	3.6 (2.8)	3.7 (2.9)	0.092
Choline (mg/day)	430.1 (151.2)	431.9 (154.3)	430.7 (151.4)	429.2 (150.2)	428.5 (149.0)	0.241
Fasting time (h)	3.6 (2.4)	3.6 (2.5)	3.6 (2.4)	3.6 (2.3)	3.6 (2.3)	0.641
eGFR (mL/min/1.73 m^2^)	91.4 (12.8)	91.1 (13.7)	91.7 (12.8)	91.4 (12.4)	91.3 (12.3)	0.639
Total choline (mmol/L)	2.5 (0.4)	2.1 (0.2)	2.4 (0.1)	2.6 (0.1)	3.0 (0.3)	< 0.001
Phosphatidylcholine (mmol/L)	2.1 (0.4)	1.7 (0.2)	2.0 (0.1)	2.2 (0.1)	2.5 (0.2)	< 0.001
Glycine (mmol/L)	0.165 (0.064)	0.157 (0.057)	0.165 (0.063)	0.169 (0.065)	0.171 (0.070)	< 0.001

*Note:* Data were reported as means (standard deviations) and No. (%) for continuous variables and categorical variables, respectively. *p* for trend across groups was computed from Spearman’s test for continuous variables and from the Mantel–Haenszel test of trend for categorical variables. MET‐min/week, metabolic equivalent of task‐minutes per week.

Abbreviations: BMI, body mass index; eGFR, estimated glomerular filtration rate.

As shown in Figure 1, initially, the Pearson correlation analysis revealed a nonsignificant negative association between dietary choline intake and plasma choline concentration (*r* = −0.0077, *p* = 0.293). However, after adjusting for age and sex, a significant positive association was observed (partial *r* = 0.0256, *p* < 0.001).

FIGURE 1Pearson and age–sex–adjusted partial Pearson correlations between dietary choline intake and circulating choline levels.

**Figure:**
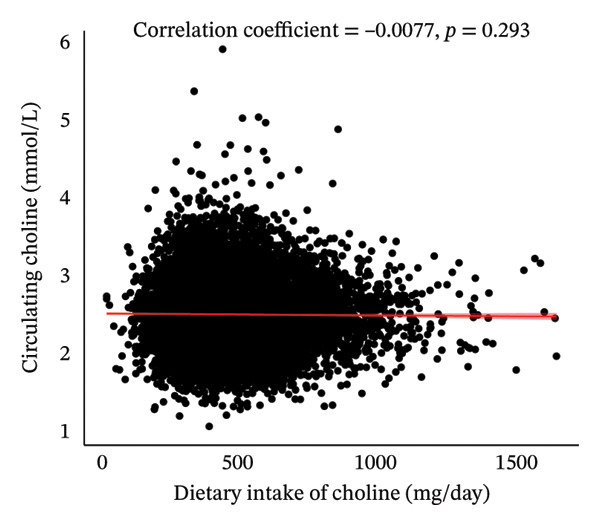
(a)

**Figure:**
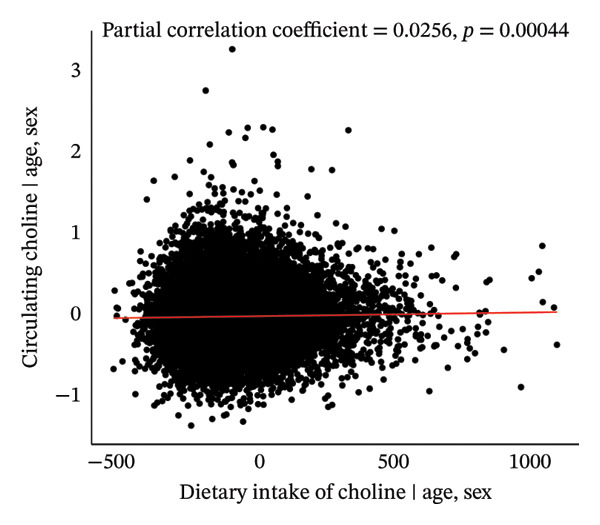
(b)

Table 2 presents the associations of circulating choline and related metabolites with BMD. In univariate models, circulating choline, phosphatidylcholines, and glycine all showed significant negative correlations with BMD. In the multivariate model, glycine remained significantly negatively associated with BMD (*β* = −0.0486, *p* = 0.004), while circulating choline demonstrated a borderline negative association (*β* = −0.0056, *p* = 0.052).

**TABLE 2:** Relationships of circulating choline and related metabolites with bone mineral density assessed by linear regression.

	Univariate model		Model 1		Model 2		Model 3		
	Coefficient (SE)	*p* value	Coefficient (SE)	*p* value	Coefficient (SE)	*p* value	Coefficient (SE)	*p* value	*p* for interaction
Choline									
Total	−0.0361 (0.0025)	< 0.001	−0.0082 (0.0026)	0.002	−0.0056 (0.0026)	0.030	−0.0056 (0.0029)	0.052	
Men	−0.0129 (0.0042)	0.002	−0.0126 (0.0042)	0.003	−0.0098 (0.0042)	0.019	−0.0097 (0.0045)	0.032	
Premenopausal women	0.0030 (0.0056)	0.593	0.0034 (0.0056)	0.543	0.0035 (0.0057)	0.533	0.008 (0.006)	0.182	0.006
Postmenopausal women	−0.0073 (0.0038)	0.056	−0.0057 (0.0039)	0.138	−0.0019 (0.0038)	0.618	−0.0052 (0.0044)	0.236	0.330
Renal function									
eGFR ≥ 90 mL/min/1.73 m^2^	−0.0298 (0.0032)	< 0.001	−0.0242 (0.0032)	< 0.001	−0.0034 (0.0033)	0.309	−0.0027 (0.0036)	0.459	
eGFR < 90 mL/min/1.73 m^2^	−0.0465 (0.0042)	< 0.001	−0.0455 (0.0042)	< 0.001	−0.0086 (0.0044)	0.050	−0.01 (0.0047)	0.034	0.006
Liver function									
ALT/AST ≤ 40 U/L	−0.0377 (0.0027)	< 0.001	−0.0349 (0.0027)	< 0.001	−0.0069 (0.0028)	0.014	−0.0059 (0.003)	0.050	
ALT/AST > 40 U/L	−0.0207 (0.0087)	0.017	−0.0162 (0.0087)	0.064	0.0065 (0.0089)	0.465	0.0034 (0.0095)	0.724	0.537
Phosphatidylcholines									
Total	−0.0382 (0.0027)	< 0.001	−0.0074 (0.0029)	0.010	−0.0043 (0.0029)	0.134	−0.0038 (0.0031)	0.217	
Men	−0.0125 (0.0046)	0.007	−0.0121 (0.0047)	0.009	−0.009 (0.0046)	0.052	−0.0089 (0.005)	0.077	
Premenopausal women	0.0061 (0.006)	0.311	0.0058 (0.0061)	0.339	0.0061 (0.0061)	0.319	0.0111 (0.0064)	0.083	0.004
Postmenopausal women	−0.0079 (0.0042)	0.061	−0.0058 (0.0042)	0.171	−0.0012 (0.0042)	0.767	−0.004 (0.0047)	0.401	0.397
Renal function									
eGFR ≥ 90 mL/min/1.73 m^2^	−0.0307 (0.0035)	< 0.001	−0.0247 (0.0035)	< 0.001	−0.0014 (0.0037)	0.693	−0.0005 (0.0039)	0.908	
eGFR < 90 mL/min/1.73 m^2^	−0.0502 (0.0046)	< 0.001	−0.0492 (0.0046)	< 0.001	−0.0078 (0.0048)	0.106	−0.0089 (0.0052)	0.084	0.005
Liver function									
ALT/AST ≤ 40 U/L	−0.0397 (0.0029)	< 0.001	−0.0369 (0.0029)	< 0.001	−0.0053 (0.0031)	0.083	−0.004 (0.0033)	0.223	
ALT/AST > 40 U/L	−0.0217 (0.0094)	0.022	−0.0161 (0.0095)	0.090	0.0084 (0.0096)	0.382	0.0049 (0.0103)	0.632	0.612
Glycine									
Total	−0.2059 (0.0152)	< 0.001	−0.0769 (0.016)	< 0.001	−0.04 (0.0162)	0.014	−0.0486 (0.0171)	0.004	
Men	−0.0987 (0.0353)	0.005	−0.1096 (0.0355)	0.002	−0.0229 (0.0364)	0.529	−0.0473 (0.0377)	0.209	
Premenopausal women	−0.0273 (0.0288)	0.342	−0.0219 (0.0287)	0.446	−0.0183 (0.0293)	0.532	−0.023 (0.0302)	0.445	0.059
Postmenopausal women	−0.0756 (0.02)	< 0.001	−0.091 (0.0199)	< 0.001	−0.0606 (0.0201)	0.003	−0.0631 (0.0214)	0.003	0.620
Renal function									
eGFR ≥ 90 mL/min/1.73 m^2^	−0.1733 (0.0198)	< 0.001	−0.1899 (0.0198)	< 0.001	−0.0387 (0.0211)	0.067	−0.0494 (0.0216)	0.022	
eGFR < 90 mL/min/1.73 m^2^	−0.2486 (0.0253)	< 0.001	−0.2659 (0.0254)	< 0.001	−0.0529 (0.0269)	0.050	−0.05 (0.0278)	0.072	0.139
Liver function									
ALT/AST ≤ 40 U/L	−0.1951 (0.0161)	< 0.001	−0.2086 (0.0161)	< 0.001	−0.0376 (0.017)	0.027	−0.0474 (0.0175)	0.007	
ALT/AST > 40 U/L	−0.3002 (0.0679)	< 0.001	−0.3146 (0.0687)	< 0.001	−0.0899 (0.0741)	0.225	−0.139 (0.0778)	0.074	0.238

*Note:* Model 1 was adjusted for age at recruitment, sex, menopausal status, race, household income per year, qualification, fasting time, smoking status, alcohol consumption, and physical activity. Model 2 was adjusted for Model 1 plus BMI, regular vitamin D and/or multiple vitamins supplement use, regular calcium supplement use, and dietary intake of energy, protein, potassium, calcium, and vitamin D. For women, hormone replacement therapy was also adjusted for. Model 3 was adjusted for Model 2 plus eGFR and plasma vitamin D level. ALT, alanine aminotransferase; AST, aspartate aminotransferase.

Abbreviation: eGFR, estimated glomerular filtration rate.

Further stratified analyses revealed significant interaction effects for circulating choline based on gender, menopausal status, and renal function. Specifically, significant differences were observed between premenopausal women and postmenopausal women, between premenopausal women and men, and between individuals with eGFR ≥ 90 mL/min/1.73 m^2^ and those with eGFR < 90 mL/min/1.73 m^2^. Notably, circulating choline was significantly negatively associated with BMD in men (*β* = −0.0097, *p* = 0.032) and in individuals with impaired renal function (*β* = −0.01, *p* = 0.034), though the associations were not significant among women and individuals with normal kidney function. However, no significant difference was found between postmenopausal women and men. When postmenopausal women and men were combined, a significant interaction effect was observed between this group and premenopausal women, as shown in Figure 2(a) (*p* for interaction = 0.010). Figure 2(b) illustrates the interaction between circulating choline and eGFR on BMD, where eGFR is treated as a continuous measure of kidney function. As eGFR decreases, the slope of the lines becomes steeper, indicating that the effect of circulating choline on BMD strengthens as kidney function declines. However, no significant interaction effect was observed when stratified by liver function.

FIGURE 2Interaction effect of sex, menopausal status, and eGFR on the relationship between circulating choline and bone mineral density. (a) The interaction effect of sex and menopausal status on the relationship between circulating choline and bone mineral density, with participants grouped into two categories: “group1” representing men and postmenopausal women and “group2” representing premenopausal women. The *p* for interaction represents the *p* value of the interaction term group ∗ circulating choline. The model was additionally adjusted for age, race, household income per year, qualification, fasting time, smoking status, alcohol consumption, physical activity, BMI, regular vitamin D and/or multiple vitamins supplement use, regular calcium supplement use, dietary intake of energy, protein, potassium, calcium, and vitamin D, eGFR, and plasma vitamin D level. For women, hormone replacement therapy was also adjusted for. (b) The interaction effect of eGFR, entered as a continuous variable, on the relationship between circulating choline and bone mineral density. The *p* for interaction represents the *p* value of the interaction term eGFR ∗ circulating choline. The model was additionally adjusted for sex, menopausal status age at recruitment, race, household income per year, qualification, fasting time, smoking status, alcohol consumption, physical activity, BMI, regular vitamin D and/or multiple vitamins supplement use, regular calcium supplement use, dietary intake of energy, protein, potassium, calcium, and vitamin D, and plasma vitamin D level. For women, hormone replacement therapy was also adjusted for. Regression lines are shown with 95% confidence intervals (shaded areas).

**Figure:**
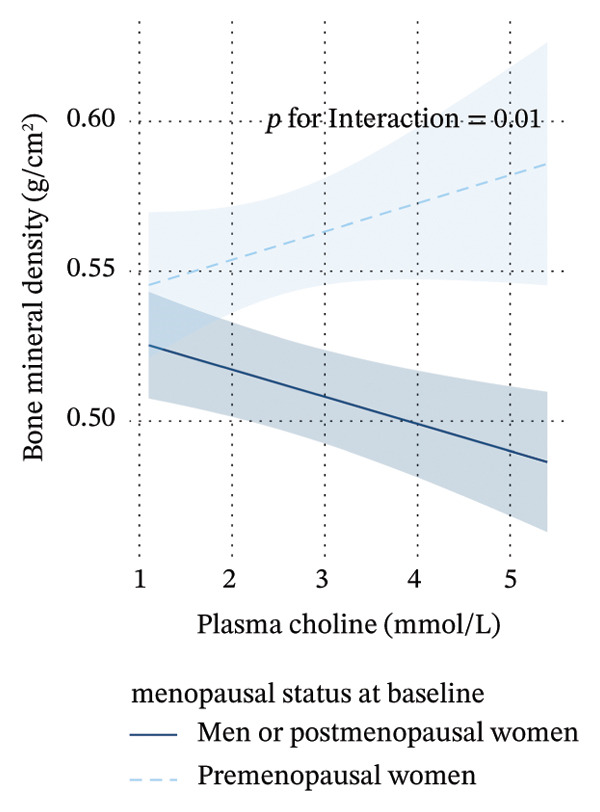
(a)

**Figure:**
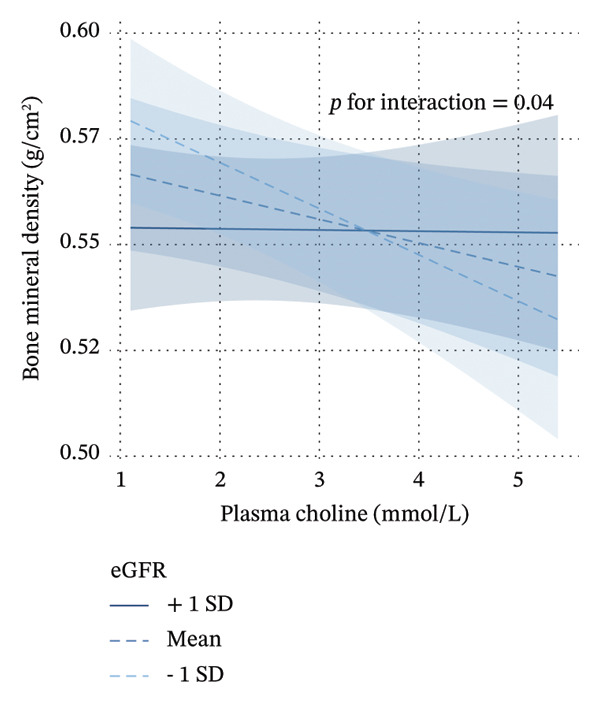
(b)

MR analyses supported a causal interpretation between circulating choline levels and BMD in men (nSNP = 49) and between glycine levels and BMD in women (nSNP = 43) (*p* < 0.05), and no heterogeneity was found (Qval > 0.05) (Figure 3). Additionally, reverse MR analysis did not provide evidence supporting an effect of BMD on the level of choline metabolites (Supporting Figure 1).

FIGURE 3Mendelian randomization results of choline metabolites and BMD. (a) Mendelian randomization results of choline metabolites and BMD in men. (b) Mendelian randomization results of choline metabolites and BMD in women.

**Figure:**
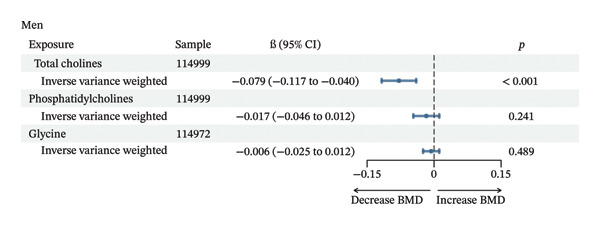
(a)

**Figure:**
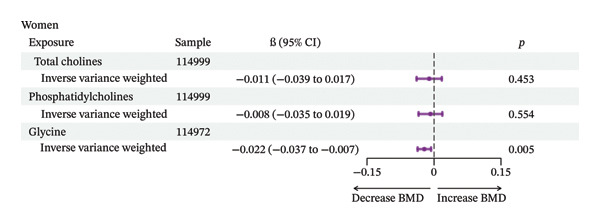
(b)

In univariate models, a positive association was observed between the intake of dietary choline and BMD in the total population (Table 3). However, after adjusting for variables, the association shifted to a nonsignificant negative correlation. Notably, although the association was not significant in participants with normal kidney function, a significant negative association was found in those with impaired kidney function (*β* = −0.0385, *p* = 0.027).

**TABLE 3:** Relationships between dietary intake of choline and bone mineral density assessed by linear regression.

	Univariate model		Model 1		Model 2		Model 3		
	Coefficient (SE)	*p* value	Coefficient (SE)	*p* value	Coefficient (SE)	*p* value	Coefficient (SE)	*p* value	*p* for interaction
Total	0.0115 (0.0065)	0.076	−0.0005 (0.0064)	0.941	−0.0156 (0.0100)	0.118	−0.0128 (0.0104)	0.218	
Men	−0.0003 (0.0095)	0.976	−0.0009 (0.0096)	0.925	−0.0291 (0.0151)	0.053	−0.0308 (0.0157)	0.050	
Premenopausal women	0.0068 (0.0145)	0.639	0.0013 (0.0147)	0.928	0.0047 (0.0232)	0.839	0.0078 (0.0240)	0.746	0.711
Postmenopausal women	−0.0038 (0.0102)	0.713	0.0046 (0.0102)	0.654	0.0065 (0.0157)	0.680	0.0188 (0.0166)	0.260	0.819
Renal function									
eGFR ≥ 90 mL/min/1.73 m^2^	0.0171 (0.0081)	0.035	0.0228 (0.0081)	0.005	−0.0023 (0.0127)	0.855	0.004 (0.0130)	0.760	
eGFR < 90 mL/min/1.73 m^2^	0.0024 (0.0115)	0.838	0.0026 (0.0115)	0.820	−0.0392 (0.0171)	0.022	−0.0385 (0.0174)	0.027	0.125
Liver function									
ALT/AST ≤ 40 U/L	0.0124 (0.0070)	0.078	0.0166 (0.0071)	0.019	−0.0136 (0.0108)	0.208	−0.0081 (0.0110)	0.462	
ALT/AST > 40 U/L	−0.0088 (0.0207)	0.672	−0.0142 (0.0209)	0.496	−0.0621 (0.0331)	0.061	−0.057 (0.0337)	0.091	0.330

*Note:* Model 1 was adjusted for age at recruitment, sex, menopausal status, race, household income per year, qualification, fasting time, smoking status, alcohol consumption, and physical activity. Model 2 was adjusted for Model 1 plus BMI, regular vitamin D and/or multiple vitamins supplement use, regular calcium supplement use, and dietary intake of energy, protein, potassium, calcium, and vitamin D. For women, hormone replacement therapy was also adjusted for. Model 3 was adjusted for Model 2 plus eGFR and plasma vitamin D level. ALT, alanine aminotransferase; AST, aspartate aminotransferase.

Abbreviation: eGFR, estimated glomerular filtration rate.

Extreme values of circulating choline, phosphatidylcholine, glycine, and heel BMD were evaluated using Tukey’s rule. This flagged 1.26% of circulating choline observations, 1.26% of phosphatidylcholine, 5.10% of glycine, and 1.99% of heel BMD values. We reran Model 3 after trimming those outliers (analytic samples ranged from 16,092 to 16,743, depending on the metabolite) and after winsorizing the same cut points while retaining the complete‐case cohort (*n* = 16,956); the direction and magnitude of the choline‐, phosphatidylcholine‐, and glycine‐BMD coefficients remained materially unchanged (Supporting Table 2).

Among Model 3 covariates, plasma vitamin D and eGFR had the highest missingness (8.4% and 4.7%, respectively), whereas all other covariates were missing in less than 0.3% of records. Consequently, Model 3 used complete‐case analysis (*n* = 16,956). To gauge potential bias from excluding participants with missing plasma vitamin D, we mean‐imputed that biomarker, appended a missing‐indicator term, and refit the model with the diet covariates retained, which increased the analytic sample to 17,705 and yielded coefficients of −0.0041 (0.0028) for circulating choline, −0.0021 (0.0030) for phosphatidylcholines, −0.0489 (0.0167) for glycine, and −0.0156 (0.0102) for dietary choline (Supporting Table 3). These results supported the robustness of the complete‐case strategy.

## 4. Discussion

Using data from the UKB, our analysis revealed a borderline significant association between circulating choline levels and BMD, as well as a significantly negative association between glycine and BMD in a middle‐aged and elderly population. Further analyses revealed that the relationship between circulating choline and BMD varied by gender, menopausal status in women, and renal function. Specifically, higher circulating choline levels were associated with lower BMD in men and individuals with impaired kidney function. Notably, among individuals with impaired renal function, a significant negative association was also observed between dietary choline intake and BMD. MR analyses further supported a potential causal interpretation of circulating choline on BMD in men and of glycine on BMD in women.

Compared to previous studies from eight European countries, where adult choline intake estimates ranged between 269 and 450 mg/day [32], our study revealed relatively comparable dietary choline intake levels, with average intakes of 386.3 and 421.2 mg/d for men and women, respectively. The results regarding the relationship between dietary choline intake and circulating choline levels have been inconsistent in previous studies. While most studies reported a positive association [14, 33, 34], a few found no substantial correlation [10, 33], and surprisingly, one study even indicated a slight negative association [33]. These discordant findings may stem from variations in measurement methods, variations in dietary questionnaires, and the adjustments made for different variables across studies. In our study, while we initially found a nonsignificant correlation between dietary choline intake and circulating choline levels, we observed a significant and weak positive correlation after controlling for age and sex. The findings suggest that exogenous food intake is an important source of the choline level in human bodies; however, an individual variability in choline metabolism exists, and circulating choline may be substantially affected by nondietary factors.

Choline’s multifaceted benefits constitute an integral part of human health, acting as a precursor for neurotransmitters and contributing to the formation of cell membranes [10]. Its implications extend to the potential prevention of various health problems, including cognitive decline, Alzheimer’s disease, and liver conditions like nonalcoholic fatty liver disease [10]. However, emerging studies have reported contradictory findings regarding the effects of choline. For instance, higher plasma choline levels have also been linked to an increased risk of atherosclerotic cardiovascular disease (ACVD) [10, 35–37]. Epidemiological studies have shown a relationship between ACVD and osteoporosis [38], as both conditions share some risk factors [39–41]. Nevertheless, whether choline or its metabolites have epigenetic implications for osteoporosis risk remains undetermined. A case–control study focusing on patients with diabetic osteoporosis found elevated levels of choline and glycine, when compared to the control group [42]. However, a study based on the National Health and Nutritional Examination Survey demonstrated that higher daily dietary choline intake was associated with a lower risk of osteoporosis, although no significant trend was observed for hip fracture risk (*p* = 0.592) [13]. Similarly, the Hordaland Health Study observed that individuals with low dietary or plasma total choline levels, particularly elderly women, had a higher risk of low femoral neck BMD [15]. Notably, our findings diverged from those of the previously mentioned studies. Our study, utilizing data from the large‐scale UKB, found no significant association between dietary choline intake and BMD in the overall population. However, there was a borderline negative association between circulating choline levels and BMD. The inconsistency in findings may be attributed to several factors. First, different assessment methods, such as dual‐energy X‐ray absorptiometry and ultrasound densitometry, were used to measure BMD. Additionally, the results may also be influenced by racial and ethnic differences in dietary habits, variations in choline‐rich food sources, and genetic variations affecting choline metabolism and gut flora as suggested by previous multiethnic studies [43]. Remarkably, previous studies have not consistently considered key factors such as menopausal status, serum vitamin D levels, physical activity, vitamin D supplement use, renal function, and dietary intake of protein and calcium, possibly introducing bias. Considering these factors is crucial when interpreting the findings and understanding the relationship between choline and osteoporosis risk.

Our study revealed significant interaction effects of gender, menopausal status, and renal function on circulating choline and BMD. A significant difference was observed between premenopausal women and a combined group of postmenopausal women and men, with a significant negative relationship between circulating choline levels and BMD among men (*β* = −0.0097, *p* = 0.032). Results from conventional observational studies may be affected by confounding factors or reverse causation, which can be mitigated to some extent through MR analysis. Our MR analyses echoed these findings, suggesting that the MR results were consistent with a possible causal effect of circulating choline on BMD in men but not in women. The negative correlation was also seen in individuals with impaired renal function (*β* = −0.01, *p* = 0.034), while such a correlation was absent in individuals with normal kidney function. A study of patients undergoing coronary angiography indicated that elevated baseline plasma choline was associated with major adverse cardiac events only when TMAO levels were also elevated. This suggested a potential link between choline and metabolic diseases was largely dependent on TMAO levels [44]. Several in vitro and in vivo studies have investigated TMAO’s effects on bone metabolism, with one revealing TMAO’s role in inhibiting osteogenesis of bone marrow mesenchymal stem cells via NF‐κB pathway activation [16], while another showed that TMAO was associated with increased ROS production, osteoclast differentiation, and bone loss through the ROS‐dependent NF‐κB signaling pathway [45]. Given the kidney’s role in TMAO elimination, renal impairment can lead to TMAO accumulation [46]. Additionally, studies have shown that estrogen deficiency increases the permeability of gut bacteria and their products, leading to immune cell activation, increased cytokine production, and, ultimately, bone loss in the bone marrow [47]. These factors may partially explain why we observed a negative association among males and individuals with impaired renal function. Therefore, our analysis suggests that higher choline levels may be associated with a higher risk of osteoporosis, at least in certain populations. It is important to note that TMAO concentrations were not available in the present UKB subset; therefore, the proposed choline–TMAO–bone pathway remains speculative in our data. Moreover, choline may influence bone biology through additional pathways, including betaine [48], phosphatidylcholine [49], and acetylcholine‐related signaling [50]. Future studies integrating TMAO and related metabolites, gut microbiome measures, and longitudinal bone outcomes are needed to clarify mechanisms.

Glycine, a nonessential amino acid, plays a pivotal role in various metabolic pathways [51]. It shares a relationship with choline through their involvement in methyl metabolism. Prior research has consistently shown a negative correlation between glycine levels and BMD [52–54]. In line with these findings, our study also observed a significant negative association between glycine and BMD, even after adjusting for potential confounding variables in the multivariate model (*β* = −0.0486, *p* = 0.004), regardless of sex and renal function. Considering that collagen, which accounts for ∼90% of bone matrix proteins, contains glycine at every third amino acid position, it is plausible that glycine metabolism is related to bone metabolic processes [55, 56].

This study has several methodological strengths, including a large, population‐based sample that enabled the inclusion of diverse populations and detailed information on diet, lifestyle, and medical history, allowing for comprehensive adjustment for confounding variables. Notably, to our knowledge, this is the first study to use MR to assess potential causal links between choline, glycine, and BMD.

However, there are several limitations to acknowledge. First, heel BMD was measured with QUS rather than DXA; however, QUS has much higher coverage in the UKB cohort than DXA, so limiting the analysis to participants with DXA data could significantly reduce the sample size and statistical power. Second, although our estimation was based on the best available evidence at the time, the reliance on the USDA database to estimate choline content may not accurately represent certain foods, and regional variations or food processing techniques could further affect choline content. Third, due to missing data (8.4% for vitamin D), Model 3 was based on complete cases. Sensitivity analyses using imputation for vitamin D increased the sample and supported the robustness of the main findings against the missing data mechanism. Fourth, we assessed multiple exposures, subgroups, and MR models without formal multiplicity corrections, so some nominal associations could reflect chance findings despite broadly consistent patterns across analyses. Fifth, residual or unmeasured confounding may persist, and the cross‐sectional design limits causal inference. Finally, while MR strengthens causal inference, its validity depends on instrumental variable assumptions that cannot be fully verified. Estimates may be biased by horizontal pleiotropy, linkage disequilibrium, or weak instruments. Furthermore, relying solely on UKB‐based GWAS resources introduces risks of sample overlap and “healthy volunteer” selection bias, which may inflate effect estimates and limit generalizability to non‐White, non‐UK populations.

Although our study identified potentially clinically meaningful associations—particularly among individuals with impaired renal function, in whom both circulating choline levels and dietary choline intake were significantly and inversely associated with BMD—these findings suggest that choline‐related metabolism may be linked to adverse bone outcomes in this population. However, despite the use of MR to strengthen causal inference, the overall evidence remains insufficient to justify changes to current dietary choline intake recommendations. Further high‐quality longitudinal studies and interventional trials are needed to confirm temporality and causality and to determine whether modifying choline intake leads to meaningful changes in bone‐related outcomes.

## 5. Conclusion

In summary, we observed modest associations between choline (and related metabolites) and calcaneal quantitative ultrasound–derived BMD, with evidence of heterogeneity across subgroups. Notably, higher choline levels were associated with lower BMD among individuals with impaired kidney function. Further well‐designed prospective longitudinal studies and interventional trials are needed to clarify temporality and strengthen causal inference.

**Nomenclature**
ACVD: Atherosclerotic cardiovascular disease
ALT: Alanine aminotransferase
AST: Aspartate aminotransferase
BMD: Bone mineral density
BMI: Body mass index
BUA: Broadband ultrasound attenuation
CKD EPI equation: Chronic kidney disease epidemiology collaboration equation
eGFR: Estimated glomerular filtration rate
GWAS: Genome wide association study
HRT: Hormone replacement therapy
MET‐min/week: Metabolic equivalent of task‐minutes per week
MR: Mendelian randomization
QC: Quality control
Scr: Blood creatinine
SNPs: Single‐nucleotide polymorphisms
SOS: Speed of sound
TMAO: Trimethylamine N‐oxide

## Author Contributions

S.C. contributed to the study’s concept and design. S.C., R.L., and Y.Z. contributed to the study’s statistical analysis. S.C. and R.L. contributed to the data interpretation and drafting of the paper. Y.L. and X.C. contributed to the data interpretation and revision of the paper. H.Y. and J.L. supervised the project.

## Funding

This project was funded by the National Natural Science Foundation of China (82172387, 82372371, 82572705), the Natural Science Foundation of Jiangsu Province (BK20230002, BK20240438), the Suzhou Science and Technology Development Plan (SKY2023035, SZM2023003), Suzhou Medical College‐QiLu Medical Research Program of Soochow University (24QL200218), and the Open Research Project of Shanghai Key Laboratory of Diabetes Mellitus (SHKLD‐KF‐2306).

## Disclosure

All authors saw and approved the final version, and no other person made a substantial contribution to the paper.

## Ethics Statement

All participants gave informed consent for data provision and linkage. UK Biobank has full ethical approval from the NHS National Research Ethics Service (16/NW/0274).

## Consent

The authors have nothing to report.

## Conflicts of Interest

The authors declare no conflicts of interest.

## Supporting Information

Calculation of dietary choline intake: Dietary choline intake was estimated using the Oxford WebQ, a 24‐h dietary assessment tool, combined with the USDA Database for the Choline Content of Common Foods. Food items were matched to choline values based on reported categories, and choline intake was calculated by multiplying food weight by its choline content. Total daily choline intake was obtained by summing all food items. The corresponding choline content of common foods, as reported in the USDA Database, is provided in Table S1.

Covariates: Baseline data on demographic, socioeconomic, and lifestyle factors (e.g., age, sex, income, education, smoking, alcohol use, and physical activity) were collected via questionnaires. Anthropometric data (e.g., BMI) and biochemical markers (e.g., ALT, AST, eGFR, and 25(OH)D) were measured using standard methods. Menopausal status, medication history, and supplement use were also recorded. Physical activity was assessed using the short International Physical Activity Questionnaire (MET‐min/week).

Supporting Table 1. Baseline characteristics of participants according to quantiles of dietary choline intake.

Supporting Table 2. Associations between circulating choline metabolites and bone mineral density after Tukey trimming and after winsorizing the same cut points within the complete‐case cohort.

Supporting Table 3. Relationships of circulating choline, related metabolites, and dietary intake of choline with bone mineral density in the plasma vitamin D missing‐indicator sensitivity analysis.

Supporting Figure 1. Mendelian randomization results for the association between the BMD and choline metabolites in men and women.

## Supporting information


**Supporting Information** Additional supporting information can be found online in the Supporting Information section.

## Data Availability

The data used in this study are available via a direct application to UK Biobank.

## References

[1] on Osteoporosis N. C. D. P. and Prevention D. , Osteoporosis Prevention, Diagnosis, and Therapy, JAMA. (2001) 285, 785–795. 11176917 10.1001/jama.285.6.785

[2] Johnell O. and Kanis J. , Epidemiology of Osteoporotic Fractures, Osteoporosis International. (2005) 16, no. S02, S3–S7, 10.1007/s00198-004-1702-6, 2-s2.0-26944458929. 15365697

[3] Cosman F. , de Beur S. J. , LeBoff M. S. et al., Clinician’s Guide to Prevention and Treatment of Osteoporosis, Osteoporosis International. (2014) 25, no. 10, 2359–2381, 10.1007/s00198-014-2794-2, 2-s2.0-84919418591. 25182228 PMC4176573

[4] Khosla S. and Hofbauer L. C. , Osteoporosis Treatment: Recent Developments and Ongoing Challenges, Lancet Diabetes & Endocrinology. (2017) 5, no. 11, 898–907, 10.1016/s2213-8587(17)30188-2, 2-s2.0-85021991961. 28689769 PMC5798872

[5] Tilman D. and Clark M. , Global Diets Link Environmental Sustainability and Human Health, Nature. (2014) 515, no. 7528, 518–522, 10.1038/nature13959, 2-s2.0-84923043971. 25383533

[6] Muñoz-Garach A. , García-Fontana B. , and Muñoz-Torres M. , Nutrients and Dietary Patterns Related to Osteoporosis, Nutrients. (2020) 12, no. 7, 10.3390/nu12071986. PMC7400143 32635394

[7] Zeisel S. H. and Da Costa K.-A. , Choline: An Essential Nutrient for Public Health, Nutrition Reviews. (2009) 67, no. 11, 615–623, 10.1111/j.1753-4887.2009.00246.x, 2-s2.0-74949113912. 19906248 PMC2782876

[8] Penry J. T. and Manore M. M. , Choline: An Important Micronutrient for Maximal endurance-Exercise Performance?, International Journal of Sport Nutrition and Exercise Metabolism. (2008) 18, no. 2, 191–203, 10.1123/ijsnem.18.2.191, 2-s2.0-44649164659. 18458362

[9] Zeisel S. H. and Niculescu M. D. , Perinatal Choline Influences Brain Structure and Function, Nutrition Reviews. (2006) 64, no. 4, 197–203, 10.1301/nr.2006.janr.197-203, 2-s2.0-33645816801. 16673755 PMC2438605

[10] Pan X.-F. , Yang J. J. , Shu X. O. et al., Associations of Circulating Choline and Its Related Metabolites With Cardiometabolic Biomarkers: an International Pooled Analysis, American Journal of Clinical Nutrition. (2021) 114, no. 3, 893–906, 10.1093/ajcn/nqab152. 34020444 PMC8408854

[11] Yang Q. , Han H. , Sun Z. et al., Association of Choline and Betaine With the Risk of Cardiovascular Disease and All-Cause Mortality: Meta-Analysis, European Journal of Clinical Investigation. (2023) 53, no. 10, 10.1111/eci.14041. 37318151

[12] Guertin K. A. , Li X. S. , Graubard B. I. et al., Serum Trimethylamine N-Oxide, Carnitine, Choline, and Betaine in Relation to Colorectal Cancer Risk in the Alpha Tocopherol, Beta Carotene Cancer Prevention Study, Cancer Epidemiology, Biomarkers & Prevention. (2017) 26, no. 6, 945–952, 10.1158/1055-9965.epi-16-0948, 2-s2.0-85020225465. PMC5608021 28077427

[13] Zhang Y.-W. , Lu P. P. , Li Y. J. et al., Low Dietary Choline Intake is Associated With the Risk of Osteoporosis in Elderly Individuals: A Population-Based Study, Food & Function. (2021) 12, no. 14, 6442–6451, 10.1039/d1fo00825k. 34076003

[14] Øyen J. , Gjesdal C. G. , Karlsson T. et al., Dietary Choline Intake is Directly Associated with Bone Mineral Density in the Hordaland Health Study, The Journal of Nutrition. (2017) 147, no. 4, 572–578, 10.3945/jn.116.243006, 2-s2.0-85020165721. 28275104

[15] Øyen J. , Nygård O. K. , Gjesdal C. G. et al., Plasma Choline, Nicotine Exposure, and Risk of Low Bone Mineral Density and Hip Fracture: The Hordaland Health Study, Journal of Bone and Mineral Research. (2014) 29, no. 1, 242–250, 10.1002/jbmr.2025, 2-s2.0-84890917102. 23794246

[16] Lin H. , Liu T. , Li X. , Gao X. , Wu T. , and Li P. , The Role of Gut Microbiota Metabolite Trimethylamine N-Oxide in Functional Impairment of Bone Marrow Mesenchymal Stem Cells in Osteoporosis Disease, Annals of Translational Medicine. (2020) 8, no. 16, 10.21037/atm-20-5307. PMC7475507 32953809

[17] Liu Y. , Guo Y. L. , Meng S. et al., Gut Microbiota–Dependent Trimethylamine N-Oxide are Related With Hip Fracture in Postmenopausal Women: A Matched Case-Control Study, Aging (Albany NY). (2020) 12, no. 11, 10633–10641, 10.18632/aging.103283. 32482913 PMC7346070

[18] Elam R. E. , Bůžková P. , Barzilay J. I. et al., Trimethylamine N-Oxide and Hip Fracture and Bone Mineral Density in Older Adults: The Cardiovascular Health Study, Bone. (2022) 161, 10.1016/j.bone.2022.116431. PMC10712255 35577327

[19] Cheng E. , Hung S.-C. , and Lin T.-Y. , Association of Trimethylamine N-Oxide and Metabolites With Kidney Function Decline in Patients With Chronic Kidney Disease, Clinical Nutrition. (2025) 44, 239–247, 10.1016/j.clnu.2024.12.001. 39709651

[20] Schwartz A. V. , Backlund J. Y. C. , de Boer I. H. et al., Risk Factors for Lower Bone Mineral Density in Older Adults With Type 1 Diabetes: A Cross-Sectional Study, Lancet Diabetes & Endocrinology. (2022) 10, no. 7, 509–518, 10.1016/s2213-8587(22)00103-6. 35576955

[21] Silva B. C. and Bilezikian J. P. , Skeletal Abnormalities in Hypoparathyroidism and in Primary Hyperparathyroidism, Reviews in Endocrine & Metabolic Disorders. (2021) 22, no. 4, 789–802, 10.1007/s11154-020-09614-0. 33200346

[22] Lademann F. , Rijntjes E. , Köhrle J. , Tsourdi E. , Hofbauer L. C. , and Rauner M. , Hyperthyroidism-Driven Bone Loss Depends on BMP Receptor Bmpr1a Expression in Osteoblasts, Communications Biology. (2024) 7, no. 1, 10.1038/s42003-024-06227-0. PMC11078941 38719881

[23] Mazziotti G. , Frara S. , and Giustina A. , Pituitary Diseases and Bone, Endocrine Reviews. (2018) 39, no. 4, 440–488, 10.1210/er.2018-00005, 2-s2.0-85055831566. 29684108

[24] Bradbury K. E. , Young H. J. , Guo W. , and Key T. J. , Dietary Assessment in UK Biobank: an Evaluation of the Performance of the Touchscreen Dietary Questionnaire, Journal of nutritional science. (2018) 7, 10.1017/jns.2017.66. PMC5799609 29430297

[25] Perez-Cornago A. , Pollard Z. , Young H. et al., Description of the Updated Nutrition Calculation of the Oxford WebQ Questionnaire and Comparison With the Previous Version Among 207,144 Participants in UK Biobank, European Journal of Nutrition. (2021) 60, no. 7, 4019–4030, 10.1007/s00394-021-02558-4. 33956230 PMC8437868

[26] Patterson K. Y. , USDA Database for the Choline Content of Common Foods, Release 2, 2008.

[27] Yao S. , Zhang M. , Dong S. S. et al., Bidirectional two-sample Mendelian Randomization Analysis Identifies Causal Associations Between Relative Carbohydrate Intake and Depression, Nature Human Behaviour. (2022) 6, no. 11, 1569–1576, 10.1038/s41562-022-01412-9. 35851841

[28] Lawlor D. A. , Harbord R. M. , Sterne J. A. , Timpson N. , and Davey Smith G. , Mendelian Randomization: Using Genes as Instruments for Making Causal Inferences in Epidemiology, Statistics in Medicine. (2008) 27, no. 8, 1133–1163, 10.1002/sim.3034, 2-s2.0-40849083720. 17886233

[29] Evans D. M. and Davey Smith G. , Mendelian Randomization: New Applications in the Coming Age of Hypothesis-Free Causality, Annual Review of Genomics and Human Genetics. (2015) 16, no. 1, 327–350, 10.1146/annurev-genom-090314-050016, 2-s2.0-84940648792. 25939054

[30] Taylor A. E. , Davies N. M. , Ware J. J. , VanderWeele T. , Smith G. D. , and Munafò M. R. , Mendelian Randomization in Health Research: Using Appropriate Genetic Variants and Avoiding Biased Estimates, Economics and Human Biology. (2014) 13, 99–106, 10.1016/j.ehb.2013.12.002, 2-s2.0-84896722557. 24388127 PMC3989031

[31] Sekula P. , Fabiola Del Greco M. , Pattaro C. , and Köttgen A. , Mendelian Randomization as an Approach to Assess Causality Using Observational Data, Journal of the American Society of Nephrology. (2016) 27, no. 11, 3253–3265, 10.1681/asn.2016010098, 2-s2.0-84992711093. 27486138 PMC5084898

[32] Vennemann F. B. , Ioannidou S. , Valsta L. M. et al., Dietary Intake and Food Sources of Choline in European Populations, British Journal of Nutrition. (2015) 114, no. 12, 2046–2055, 10.1017/s0007114515003700, 2-s2.0-84949319346. 26423357

[33] Edwards C. G. , Walk A. M. , Thompson S. V. et al., Dietary Lutein Plus Zeaxanthin and Choline Intake is Interactively Associated With Cognitive Flexibility in Middle-Adulthood in Adults With Overweight and Obesity, Nutritional Neuroscience. (2022) 25, no. 7, 1437–1452, 10.1080/1028415x.2020.1866867. 33448903

[34] Fischer L. M. , da Costa K. A. , Galanko J. et al., Choline Intake and Genetic Polymorphisms Influence Choline Metabolite Concentrations in Human Breast Milk and Plasma, American Journal of Clinical Nutrition. (2010) 92, no. 2, 336–346, 10.3945/ajcn.2010.29459, 2-s2.0-77955496122. 20534746 PMC2904035

[35] Zuo H. , Svingen G. F. T. , Tell G. S. et al., Plasma Concentrations and Dietary Intakes of Choline and Betaine in Association With Atrial Fibrillation Risk: Results from 3 Prospective Cohorts With Different Health Profiles, Journal of the American Heart Association. (2018) 7, no. 8, 10.1161/jaha.117.008190, 2-s2.0-85045345306. PMC6015426 29650710

[36] Roe A. J. , Zhang S. , Bhadelia R. A. et al., Choline and Its Metabolites are Differently Associated With Cardiometabolic Risk Factors, History of Cardiovascular Disease, and MRI-Documented Cerebrovascular Disease in Older Adults, American Journal of Clinical Nutrition. (2017) 105, no. 6, 1283–1290, 10.3945/ajcn.116.137158, 2-s2.0-85020542910. 28356272 PMC5445668

[37] Fu B. C. , Hullar M. A. , Randolph T. W. et al., Associations of Plasma Trimethylamine N-Oxide, Choline, Carnitine, and Betaine with Inflammatory and Cardiometabolic Risk Biomarkers and the Fecal Microbiome in the Multiethnic Cohort Adiposity Phenotype Study, American Journal of Clinical Nutrition. (2020) 111, no. 6, 1226–1234, 10.1093/ajcn/nqaa015. 32055828 PMC7266689

[38] Crepaldi G. and Maggi S. , Epidemiologic Link Between Osteoporosis and Cardiovascular Disease, Journal of Endocrinological Investigation. (2009) 32, 2–5. 19724158

[39] Lampropoulos C. E. , Papaioannou I. , and D’cruz D. P. , Osteoporosis—A Risk Factor for Cardiovascular Disease?, Nature Reviews Rheumatology. (2012) 8, no. 10, 587–598, 10.1038/nrrheum.2012.120, 2-s2.0-84867068673. 22890244

[40] Jeon Y. K. , Lee J. G. , Kim S. S. et al., Association Between Bone Mineral Density and Metabolic Syndrome in Pre-and Postmenopausal Women, Endocrine Journal. (2011) 58, no. 2, 87–93, 10.1507/endocrj.k10e-297, 2-s2.0-79952732068. 21242648

[41] Szulc P. , Varennes A. , Delmas P. D. , Goudable J. , and Chapurlat R. , Men With Metabolic Syndrome Have Lower Bone Mineral Density but Lower Fracture Risk—The MINOS Study, Journal of Bone and Mineral Research. (2010) 25, no. 6, 1446–1454, 10.1002/jbmr.13, 2-s2.0-77953498518. 20200928

[42] Liang W.-D. et al., Metabolomics and Its Application in the Mechanism Analysis on Diabetic Bone Metabolic Abnormality, European Review for Medical and Pharmacological Sciences. (2020) 24. 10.26355/eurrev_202009_23047 33015802

[43] Yonemori K. M. , Lim U. , Koga K. R. et al., Dietary Choline and Betaine Intakes Vary in an Adult Multiethnic Population, Journal of Nutrition. (2013) 143, no. 6, 894–899, 10.3945/jn.112.171132, 2-s2.0-84878228601. 23616508 PMC3652885

[44] Wang Z. , Tang W. H. W. , Buffa J. A. et al., Prognostic Value of Choline and Betaine Depends on Intestinal Microbiota-Generated Metabolite Trimethylamine-N-Oxide, European Heart Journal. (2014) 35, no. 14, 904–910, 10.1093/eurheartj/ehu002, 2-s2.0-84898859680. 24497336 PMC3977137

[45] Wang N. , Hao Y. , and Fu L. , Trimethylamine-N-Oxide Promotes Osteoclast Differentiation and Bone Loss via Activating ROS-Dependent NF-κB Signaling Pathway, Nutrients. (2022) 14, no. 19, 10.3390/nu14193955. PMC9573743 36235607

[46] Canyelles M. , Borràs C. , Rotllan N. , Tondo M. , Escolà-Gil J. C. , and Blanco-Vaca F. , Gut microbiota-derived TMAO: A Causal Factor Promoting Atherosclerotic Cardiovascular Disease?, International Journal of Molecular Sciences. (2023) 24, no. 3, 10.3390/ijms24031940. PMC9916030 36768264

[47] Li J.-Y. , Chassaing B. , Tyagi A. M. et al., Sex Steroid Deficiency–Associated Bone Loss is Microbiota Dependent and Prevented by Probiotics, Journal of Clinical Investigation. (2016) 126, no. 6, 2049–2063, 10.1172/jci86062, 2-s2.0-84974527504. 27111232 PMC4887186

[48] Villa I. , Senesi P. , Montesano A. et al., Betaine Promotes Cell Differentiation of Human Osteoblasts in Primary Culture, Journal of Translational Medicine. (2017) 15, no. 1, 10.1186/s12967-017-1233-5, 2-s2.0-85020234196. PMC5463390 28592272

[49] Fu M. , Tian Y. , Zhang T. , Zhan Q. , Zhang L. , and Wang J. , Comparative Study of DHA-Enriched Phosphatidylcholine and EPA-Enriched Phosphatidylcholine on Ameliorating High Bone Turnover via Regulation of the Osteogenesis-Related Wnt/β-Catenin Pathway in Ovariectomized Mice, Food & Function. (2020) 11, 10094–10104, 10.1039/d0fo01563f. 33140795

[50] Li J. , Zhang Z. , Tang J. , Hou Z. , Li L. , and Li B. , Emerging Roles of Nerve-Bone Axis in Modulating Skeletal System, Medicinal Research Reviews. (2024) 44, no. 4, 1867–1903, 10.1002/med.22031. 38421080

[51] Razak M. A. , Begum P. S. , Viswanath B. , and Rajagopal S. , Multifarious Beneficial Effect of Nonessential Amino Acid, Glycine: a Review, Oxidative Medicine and Cellular Longevity. (2017) 2017, no. 1, 10.1155/2017/1716701, 2-s2.0-85015761051. PMC5350494 28337245

[52] Moayyeri A. , Cheung C. L. , Tan K. C. et al., Metabolomic Pathways to Osteoporosis in Middle-Aged Women: A Genome-Metabolome-Wide Mendelian Randomization Study, Journal of Bone and Mineral Research. (2018) 33, no. 4, 643–650, 10.1002/jbmr.3358, 2-s2.0-85045109546. 29232479 PMC5972819

[53] Eriksson A. L. , Serum Glycine Levels are Associated With Cortical Bone Properties and Fracture Risk in Men, Journal of Clinical Endocrinology & Metabolism. (2021) 106, e5021–e5029. 34297085 10.1210/clinem/dgab544

[54] Zhang X. , Xu H. , Li G. H. et al., Metabolomics Insights into Osteoporosis Through Association With Bone Mineral Density, Journal of Bone and Mineral Research. (2020) 36, no. 4, 729–738, 10.1002/jbmr.4240. PMC8488880 33434288

[55] Horng J.-C. , Kotch F. W. , and Raines R. T. , Is Glycine a Surrogate for Ad-Amino Acid in the Collagen Triple Helix?, Protein Science. (2007) 16, no. 2, 208–215, 10.1110/ps.062560107, 2-s2.0-33846492392. 17189476 PMC2203290

[56] Morgan E. , Barnes G. , and Einhorn T. , Marcus R. , Feldman D. , Nelson D. A. , and Rosen C. J. , The Bone Organ System: Form and Function-, Osteoporosis, 2008.

