# Supplementary material for: Associations of Dietary and Circulating Choline and Glycine With Bone Mineral Density: UK Biobank Analyses With Mendelian Randomization
Source: Int J Endocrinol. 2026 Apr 10;2026:2237464. doi: 10.1155/ije/2237464 (PMC13069184; doi:10.1155/ije/2237464)
Supplement: Supplementary file 1 — Supporting Information Additional supporting information can be found online in the Supporting Information section. [file IJE-2026-2237464-s001.docx]

# Supplemental Files

# Supplementary Methods

**Calculation of dietary choline intake**

The UK Biobank study used the Oxford WebQ, a web-based self-administered 24-hour dietary assessment tool, to collect detailed dietary data. To evaluate choline intake, we applied a comprehensive food grouping system developed for the database, and used the USDA Database for Choline Content of Common Foods to supplement our data. We acknowledged the potential variations in choline content due to recipe differences and differences in choline content between individual ingredients in multi-component foods. For meat and fish, we used the average choline value for uncooked meats and fishes (80 mg total choline / 100 g), as no information was available for specific parts or preparations. For other foods, we used the closest match available on the database or the average values for the reported categories (30 mg total choline / 100 g of vegetables and fruits; 6.5 mg total choline / 100 g of fats and oils) if no close match was found. For beverages, we converted milliliters to grams using the appropriate conversion factor. A second researcher (XC) reviewed all the food matching and further modifcations were made after discussion with the other researchers. The corresponding choline content of common foods, as reported in the USDA Database, is provided in **Table S1**. Finally, the choline intake for each food item was calculated by multiplying its choline value by the reported food weight in the WebQ, and the total choline intake for the day was obtained by summing up these values.

**Table S1. Choline Content of Common Foods Based on USDA Database**

| Name | Includes | Corresponding USDA food | Choline Content (mg choline / 100 g of food) |
| --- | --- | --- | --- |
| 1) Cereals & cereal products |  |  |  |
| White bread | White bread, sliced, baguette, bap, roll | Bread, white, commercially crumbs) prepared, (includes soft bread) | 15 |
| Wholemeal bread | Wholemeal bread, sliced, baguette, bap, roll | Bread, whole-wheat, commercially prepared | 27 |
| Mixed bread, brown & seeded | Mixed, brown or seeded bread, sliced, baguette, bap, roll | Bread, whole-wheat, commercially prepared | 27 |
| Other bread | Naan, garlic bread, other bread (including gluten free) | Bread, french or vienna (includes sourdough) | 15 |
| Savoury crackers | Oatcakes, crispbreads (including gluten free) | Crackers, standard snack-type, regular | 14 |
| Bran cereal | Bran cereal | Cereals ready-to-eat, KRAFT, POST Raisin Bran Cereal | 29 |
| Biscuit cereal | Wholewheat biscuit cereal | Cereals ready-to-eat, RALSTON Corn Biscuits | 11 |
| Oat cereal (non-sugar) | Porridge oats (including milk/dried fruit added) | Cereals, oats, regular and quick and instant, unenriched, cooked with water, without salt | 32 |
| Oat cereal (sugar) | Oatcrunch breakfast cereal | Cereals ready-to-eat, QUAKER, QUAKER 100% Natural Cereal with oats, honey, and raisins | 32 |
| Muesli | Muesli (with or without dried fruit) | Cereals ready-to-eat, QUAKER, QUAKER 100% Natural Cereal with oats, honey, and raisins | 32 |
| Other cereal (sugar) | Plain and sugary breakfast cereal (with/without dried fruit) | Cereals ready-to-eat, UNCLE SAM CEREAL | 50 |
| White pasta & rice | White pasta, rice, couscous, gluten free pasta | Rice, white, long grain, regular, cooked | 2.1 |
| Wholemeal pasta, brown rice & other wholegrains | Brown and wholemeal pasta and rice | Wheat flour, whole-grain | 31 |
| 2) Mixed-dishes |  |  |  |
| Pizza | Pizza (including gluten free crust) | Pizza chain, 14" pizza, cheese topping, regular crust | 16 |
| Grain dishes - added fat | Double and single crust pies, crumble pies, Yorkshire pudding, snackpot noodles | Pancakes, plain, dry mix, complete, prepared | 19 |
| Samosa, pakora | Indian samosa, pakora snacks | Fast foods, nachos, with cheese, beans, ground beef, and peppers | 28 |
| Soups | Soups, homemade, powdered and canned | Soup, tomato, canned, condensed | 13 |
| Sushi | Sushi | Rice, white, long grain, regular, cooked | 2.1 |
| 3) Dairy & dairy-free products |  |  |  |
| Whole milk | Whole milk >3.6 g fat per 100 g (cow, goat, sheep) | Milk, whole, 3.25% milkfat | 14 |
| Semi-skimmed milk | Semi-skimmed milk >1 g fat per 100 g (cow, other) | Milk, reduced fat fluid, 2% milk fat, with added vitamin A | 16 |
| Skimmed milk | Skimmed milk <1 g fat per 100 g (cow, cholesterol lowering, powdered) | Milk, low fat, fluid, 1% milkfat, with added vitamin A | 18 |
| Rice/oat drink | Rice and oat vegetable drinks | Cereals, QUAKER, corn grits, instant, plain, prepared with water | 2.6 |
| Soy drink | Soya drinks (including calcium fortified) | Soy milk, fluid | 24 |
| Full fat yogurt | Whole milk yogurt (plain) | Frozen yogurts, flavors other than chocolate | 23 |
| Low fat yogurt | Fat free and lower fat yogurt, plain or flavoured | Yogurt, plain, low fat, 12 grams protein per 8 ounces | 15 |
| High fat cheese | Cheese >17.5 g fat per 100 g, including hard cheese, soft cheese, spreadable, Blue, Feta, Mozzarella, Goats, other) | Cheese, cheddar | 17 |
| Medium & low fat cheese | Cheese <=17.5g fat per 100 g, including hard and spreadable lower fat cheese, Cottage | Cheese, cottage, lowfat, 2% milk fat | 16 |
| Cream | Cream (cow’s milk) | Cheese, cream | 27 |
| 4) Egg & egg dishes |  |  |  |
| Egg and egg dishes | Whole eggs and processed (omelette, scotch eggs, other) | Whole eggs | 250 |
| 5) Fat & spreads |  |  |  |
| Olive oil (drizzling/dunking) | Olive oil | Oil, olive, salad or cooking | 0.3 |
| Dairy fat spread lower fat | Spreadable/lower fat butter, dairy-based very low fat spread | Butter, with salt | 19 |
| Dairy fat spread | Spreadable normal fat butter, dairy-based normal fat spread (including cholesterol lowering spread) | Butter, with salt | 19 |
| Vegetable spread lower fat | Olive oil based lower fat spread, plant-based lower fat margarine and soya-based lower fat spread (including cholesterol lowering spread) | Margarine, vegetable oil spread, 60% fat, tub/bottle | 10 |
| Vegetable spread | Olive oil based spread, plant-based soft or hard margarine and soya-based spread (including cholesterol lowering spread) | Margarine, 70% vegetable oil spread, soybean and soybean (hydrogenated) | 14 |
| 6) Meat & meat products |  |  |  |
| Poultry | Poultry (with/without skin) | Meats and fishes | 80 |
| Pork | Pork | Meats and fishes | 80 |
| Beef | Beef | Meats and fishes | 80 |
| Lamb | Lamb | Meats and fishes | 80 |
| Other meat, offal | Other meat including offal | Meats and fishes | 80 |
| Processed meat | Sausages, bacon (with and without fat), ham, liver pate | Meats and fishes | 80 |
| Breaded/battered Chicken | Fried poultry with batter/breadcrumbs | Meats and fishes | 80 |
| 7) Fish & fish dishes |  |  |  |
| White fish & tinned tuna | Tinned tuna, white fish, other fish | Meats and fishes | 80 |
| Shellfish | Prawns, lobster, crab, shellfish | Meats and fishes | 80 |
| Oily fish | Oily fish, including salmon, | Meats and fishes | 80 |
| Breaded/battered Fish | Fried fish with batter/breadcrumbs | Meats and fishes | 80 |
| 8) Meat substitutes |  |  |  |
| Vegetarian meals | Quorn-based and vegetarian burgers and products | Meat substitutes - vegetarian | 19 |
| Soy-based meals | Tofu-based products | Tofu, soft, prepared with calcium sulfate and magnesium chloride (nigari) | 27 |
| 9) Vegetables & potatoes |  |  |  |
| Raw salad | Mixed side salad, lettuce, watercress | Vegetables and fruits | 30 |
| Green leafy/cabbages | Broccoli, cabbage, kale, cauliflower, spinach, sprouts | Cabbage, raw | 11 |
| Root vegetables | Beetroot, carrots, celery, parsnip, turnip | Carrots, raw | 8.8 |
| Tomatoes | Fresh and tinned tomatoes | Tomato products, canned, paste, without salt added | 39 |
| Allium vegetables | Garlic, leek, onion | Onions, raw | 6.1 |
| Other vegetables (mushrooms, fruiting, mixed) | Mushrooms, mixed vegetables, avocado, broad beans, green beans, butternut squash, courgettes, peppers, other | Vegetables and fruits | 30 |
| Peas/sweetcorn | Peas, sweetcorn | Peas, green, frozen, cooked, boiled, drained without salt | 28 |
| Potatoes/sweet potatoes (baked/boiled) | Potatoes, sweet potatoes, boiled or baked | Sweet potato, cooked, baked in skin, without salt | 13 |
| Mashed potatoes | Potatoes, mashed | Potatoes, mashed, home-prepared, whole milk added | 14 |
| Fried/roast potatoes | Potatoes and chips, fried or roasted with fat | Potatoes, french fried, frozen, home prepared, heated in oven without salt | 24 |
| Legumes/pulses | Baked beans, pulses | Beans, baked, canned, plain or vegetarian | 32 |
| Vegetable side dishes | Coleslaw, salad with added fat/mayonnaise | Vegetables and fruits | 30 |
| Vegetable dips | Hummus, guacamole | Vegetable dips | 19 |
| 10) Fruits |  |  |  |
| Citrus | Grapefruit, orange, satsuma | Oranges, raw, navel | 8.4 |
| Berries | Blackberries, strawberries, blueberries, raspberries, cherries | Blackberries, raw | 8.6 |
| Apples & pears | Apples and pears | Apples, raw, with skin | 3.4 |
| Bananas & other fruit | Bananas, mixed fruit, grapes, mango, melon, peach, pineapple, kiwi, other | Other fruit | 30 |
| Dried fruit | Dried fruit, prunes | Plums, dried, (prunes), uncooked | 10 |
| Stewed fruit | Stewed fruit, plums | Vegetables and fruits | 30 |
| 11) Nuts & seeds |  |  |  |
| Salted nuts & seeds | Salted peanuts and nuts | Nuts, almonds | 52 |
| Unsalted nuts & seeds | Unsalted peanuts and nuts | Nuts, almonds | 52 |
| 12) Sugar, preserves, cakes & confectionery, snacks |  |  |  |
| Added sugars & preserves | Table sugar, honey, jam and preserves | Sugars, brown | 2.5 |
| Chocolate confectionery | Chocolate bar (including white, milk and dark chocolate), chocolate-covered raisins, chocolate-covered sweets | Baking chocolate, unsweetened, squares | 46 |
| Other sweets | Hard and soft sweets (including sugar free) | Candies, caramels, chocolate-flavor roll | 19 |
| Savoury snacks | Crisps, savoury biscuits, cheese snacks, other savoury biscuits | Snacks, corn-based, extruded, puffs or twists, cheese-flavor | 12 |
| Biscuits | Chocolate biscuits, plain biscuits, sweet biscuits and cookies | Biscuits, plain or buttermilk, commercially baked | 8.9 |
| Milk-dairy desserts | Ice cream, milk puddings, milk-based desserts, cheesecake | Fast foods, chocolate, dairy dessert | 28 |
| Desserts & cakes & pastries | Pancakes, croissant, Danish pastries, scones, fruitcakes, cakes, doughnuts, sponge puddings, other desserts, cereal bars, sweet snacks | Pancakes, plain, dry mix, complete, prepared | 19 |
| Soya-based desserts & yogurt | Soya-based desserts | Soy milk, fluid | 24 |
| Nut-based spreads | Peanut-butter and chocolate-based spread | Peanut butter, smooth style with salt | 66 |
| 13) Sauces & condiments |  |  |  |
| Sauces (higher fat) | Mayonnaise, salad dressing, pesto, cheese sauce, white sauce, gravy | Sauce, salsa, ready to serve | 12 |
| Sauces (lower fat) | Yeast, chutney, olives, ketchup, brown sauce, tomato sauce | Tomato products, canned, sauce | 9.9 |
| 14) Non-alcoholic beverages* |  |  |  |
| Fruit juice | Orange, grapefruit drink and 100% fruit juice | Apple juice, calcium enriched with added vitamin C | 1.8 |
| Coffee, caffeinated | Normal instant, filter, cappuccino, espresso coffee | Coffee, brewed from grounds, prepared with tap water | 2.6 |
| Coffee, decaffeinated | Decaffeinated instant, filter, cappuccino, espresso coffee | Coffee, brewed from grounds, prepared with tap water | 2.6 |
| Tea | Black, green and other tea | Iced tea, fast food, unsweetened | 0.4 |
| Tea, decaffeinated | Decaffeinated black, herbal tea, rooibos | Iced tea, fast food, unsweetened | 0.4 |
| SSBs & other sugary drinks | Fizzy sugary drinks, squash, fruit smoothies | Carbonated beverage, orange | 0.6 |
| Low/non sugar SSBs | Low calorie fizzy drinks and squash | Carbonated beverage, low calorie, cola or pepper-type, with aspartame, contains caffeine | 0 |
| Water/sparkling water | Plain water, sparkling water |  | 0 |
| Milk-based & powdered drinks | Dairy-based smoothies, milk-based drinks, hot chocolate | Shake, fast food, vanilla | 22 |
| 15) Alcoholic beverages* |  |  |  |
| White wine | White wine | Alcoholic beverage, wine table, white | 4.7 |
| Red wine | Red and rose wine | Alcoholic beverage, wine, table, red | 5.7 |
| Fortified wine | Fortified wine | Alcoholic beverage, wine table, white | 4.7 |
| Beer & Cider | Beer and cider | Alcoholic beverage, beer, regular | 9.9 |
| Spirits | Spirits and other alcoholic drinks | Alcoholic beverage, wine table, white | 4.7 |

## Covariates

Data on demographic, socioeconomic, and lifestyle factors were collected at baseline through questionnaires. Demographic information, such as sex, age, race(White, non-White), annual household income(<£18,000, £18,000–30,999, £31,000–51,999, £52,000–100,000, >£100,000, do not know, prefer not to answer), qualification(higher degree [college or university degree, or professional qualifications], school degree [A levels, AS levels, O levels, GCSEs, or CSEs], vocational qualifications [NVQ, HND, or HNC], other [none of the above]), fasting time, smoking status (never, former, current, prefer not to answer), alcohol consumption (never, former, current, prefer not to answer), and physical activity, were collected through a self-completed touch-screen questionnaire.

Physical activity was assessed using the Short International Physical Activity Questionnaire and recorded in Metabolic Equivalent of Task-minutes per week (MET-min/week). Menopausal status, medication history (for women hormone-replacement therapy), and supplement use (including calcium supplement, vitamin D, and/or multiple vitamins supplement) were determined using a self-reported questionnaire and nurse’s interview. Body mass index (BMI) was calculated as weight (kg) divided by the square of height (m). Fasting time was recorded as the interval between consumption of food or drink and blood sample(s) being taken. Alanine aminotransferase (ALT), aspartate aminotransferase (AST), and blood creatinine (Scr) levels were measured by the Beckman Coulter AU5800 system through IFCC analysis and enzymatic analysis methods, respectively. Estimated glomerular filtration rate (eGFR) was calculated by CKD EPI Equation: GFR = 141 × min (Scr /κ, 1)^α^ × max(Scr /κ, 1)^-1.209^ × 0.993^Age^ × 1.018 [if female] × 1.159 [if black]), κ = 0.7 (females) or 0.9 (males), α = -0.329 (females) or -0.411 (males)^1^. Serum concentrations of 25(OH)D were measured using a direct competitive chemiluminescent immunoassay method by DiaSorin Liaison XL Analyzer (Diasorin S.p.A). Further information is available on the UK-Biobank Web site (https://www.ukbiobank.ac.uk).

**References**

1. Levey, A. S. *et al.* A new equation to estimate glomerular filtration rate. *Annals of internal medicine* **150**, 604–612 (2009).

**Supplementary Table**

**Supplementary Table 1. Baseline characteristics of participants according to quantiles of dietary choline intake**

|  | **Total** | **Dietary choline intake level** | | | | |
| --- | --- | --- | --- | --- | --- | --- |
|  |  | **Q1** | **Q2** | **Q3** | **Q4** | **P for trend** |
| N | 18769 | 4693 | 4692 | 4692 | 4692 |  |
| Age, years | 55.7 (7.9) | 54.7 (8.0) | 55.6 (7.8) | 56.4 (7.8) | 56.1 (7.9) | <0.001 |
| White race | 17340 (92.6%) | 4263 (91.1%) | 4355 (93.0%) | 4396 (93.9%) | 4326 (92.5%) | 0.004 |
| Male | 9126 (48.6%) | 1977 (42.1%) | 2100 (44.8%) | 2384 (50.8%) | 2665 (56.8%) | <0.001 |
| Menopausal status |  |  |  |  |  | <0.001 |
| *Premenopausal* | 3201 (33.2%) | 1024 (37.7%) | 858 (33.1%) | 711 (30.8%) | 608 (30.0%) |  |
| *Postmenopausal* | 6442 (66.8%) | 1692 (62.3%) | 1734 (66.9%) | 1597 (69.2%) | 1419 (70.0%) |  |
| Average income per year, £ |  |  |  |  |  | 0.006 |
| *<18000* | 2236 (11.9%) | 545 (11.6%) | 495 (10.5%) | 574 (12.2%) | 622 (13.3%) |  |
| *18000-30999* | 4010 (21.4%) | 986 (21.0%) | 979 (20.9%) | 1021 (21.8%) | 1024 (21.8%) |  |
| *31000-51999* | 4973 (26.5%) | 1227 (26.1%) | 1281 (27.3%) | 1260 (26.9%) | 1205 (25.7%) |  |
| *52000-99999* | 4446 (23.7%) | 1127 (24.0%) | 1194 (25.4%) | 1047 (22.3%) | 1078 (23.0%) |  |
| *≥100000* | 1343 (7.2%) | 359 (7.6%) | 341 (7.3%) | 320 (6.8%) | 323 (6.9%) |  |
| Qualifications |  |  |  |  |  | 0.001 |
| *Higher degree* | 11524 (61.6%) | 2821 (60.3%) | 3000 (64.1%) | 2929 (62.6%) | 2774 (59.3%) |  |
| *Any school degree* | 5036 (26.9%) | 1340 (28.6%) | 1216 (26.0%) | 1211 (25.9%) | 1269 (27.1%) |  |
| *Vocational qualifications* | 830 (4.4%) | 185 (4.0%) | 186 (4.0%) | 210 (4.5%) | 249 (5.3%) |  |
| *Other* | 1329 (7.1%) | 332 (7.1%) | 280 (6.0%) | 329 (7.0%) | 388 (8.3%) |  |
| Smoking status |  |  |  |  |  | <0.001 |
| *Never* | 10747 (57.4%) | 2785 (59.6%) | 2786 (59.5%) | 2687 (57.3%) | 2489 (53.2%) |  |
| *Previous* | 6685 (35.7%) | 1562 (33.4%) | 1600 (34.2%) | 1702 (36.3%) | 1821 (38.9%) |  |
| *Current* | 1293 (6.9%) | 326 (7.0%) | 298 (6.4%) | 298 (6.4%) | 371 (7.9%) |  |
| Drinking status |  |  |  |  |  | <0.001 |
| *Never* | 468 (2.5%) | 168 (3.6%) | 110 (2.3%) | 101 (2.2%) | 89 (1.9%) |  |
| *Previous* | 490 (2.6%) | 162 (3.5%) | 113 (2.4%) | 106 (2.3%) | 109 (2.3%) |  |
| *Current* | 17803 (94.9%) | 4360 (93.0%) | 4468 (95.2%) | 4483 (95.6%) | 4492 (95.8%) |  |
| Physical activity, MET-min/week |  |  |  |  |  | <0.001 |
| *<600* | 2967 (15.8%) | 861 (18.3%) | 768 (16.4%) | 688 (14.7%) | 650 (13.9%) |  |
| *600-2999* | 8898 (47.4%) | 2197 (46.8%) | 2276 (48.5%) | 2258 (48.1%) | 2167 (46.2%) |  |
| *≥3000* | 4270 (22.8%) | 935 (19.9%) | 967 (20.6%) | 1102 (23.5%) | 1266 (27.0%) |  |
| BMI, kg/m^2^ | 26.6 (4.4) | 26.3 (4.4) | 26.3 (4.2) | 26.5 (4.2) | 27.2 (4.5) | <0.001 |
| Regular vitamin D supplements use | 4630 (24.7%) | 1159 (24.7%) | 1152 (24.6%) | 1135 (24.3%) | 1184 (25.3%) | 0.612 |
| Regular calcium supplements use | 1302 (6.9%) | 324 (6.9%) | 327 (7.0%) | 331 (7.1%) | 320 (6.8%) | 0.919 |
| Hormone-replacement therapy | 3096 (32.1%) | 788 (29.0%) | 827 (32.0%) | 785 (34.1%) | 696 (34.4%) | <0.001 |
| Daily dietary intake |  |  |  |  |  |  |
| Energy, kJ | 8713.0 (2353.2) | 7078.5 (1804.6) | 8270.8 (1740.0) | 9114.7 (1934.7) | 10388.2 (2507.9) | <0.001 |
| Protein, g | 81.3 (23.5) | 61.0 (15.2) | 76.1 (14.3) | 85.8 (16.0) | 102.4 (24.9) | <0.001 |
| Potassium, mg | 3690.6 (1025.1) | 2813.8 (684.1) | 3520.7 (661.1) | 3954.7 (767.6) | 4473.5 (1108.6) | <0.001 |
| Calcium, mg | 984.3 (327.6) | 816.8 (281.8) | 951.0 (281.1) | 1037.5 (298.6) | 1132.1 (357.6) | <0.001 |
| Vitamin D, ug | 3.7 (2.9) | 2.4 (2.1) | 3.2 (2.4) | 3.8 (2.6) | 5.4 (3.4) | <0.001 |
| Choline, mg | 403.3 (147.5) | 247.9 (46.9) | 343.0 (20.7) | 422.4 (26.5) | 599.7 (130.4) | <0.001 |
| Fasting time, hours | 3.6 (2.4) | 3.7 (2.6) | 3.5 (2.2) | 3.5 (2.3) | 3.6 (2.4) | 0.007 |
| Vitamin D, nmol/L | 48.3 (20.3) | 46.6 (20.3) | 48.0 (20.4) | 49.0 (20.0) | 49.5 (20.5) | <0.001 |
| eGFR, mL/min/1.73m^2^ | 91.4 (12.8) | 92.1 (13.0) | 91.3 (12.8) | 90.9 (12.6) | 91.2 (12.9) | <0.001 |
| Total choline, mmol/l | 2.5 (0.4) | 2.5 (0.4) | 2.6 (0.4) | 2.5 (0.4) | 2.5 (0.4) | 0.081 |
| Phosphatidylcholine, mmol/l | 2.1 (0.4) | 2.1 (0.4) | 2.1 (0.4) | 2.1 (0.4) | 2.1 (0.4) | 0.146 |
| Glycine, mmol/l | 0.165 (0.064) | 0.174 (0.068) | 0.168 (0.065) | 0.163 (0.062) | 0.156 (0.059) | <0.001 |

Abbreviations:BMI,body mass index;eGFR,estimated glomerular filtration rate;MET-min/week,metabolic equivalent of task-minutes per week.

Data were reported as means (standard deviations) and No. (%) for continuous variables and categorial variables, respectively.

P for trend across groups was computed from Spearman test for continuous variables and from Mantel-Haenszel test of trend for categorical variables.

**Supplementary Table 2. Associations between circulating choline metabolites and bone mineral density after Tukey trimming and after winsorizing the same cut points within the complete-case cohort**

| Dataset | N | Coefficient (SE) | P value |
| --- | --- | --- | --- |
| **Choline** |  |  |  |
| Primary | 16,956 | -0.0056 (0.0029) | 0.050 |
| Trimmed | 16,743 | -0.0053 (0.0031) | 0.086 |
| Winsorized | 16,956 | -0.0056 (0.0029) | 0.058 |
| **Phosphatidylcholines** |  |  |  |
| Primary | 16,956 | -0.0038 (0.0031) | 0.209 |
| Trimmed | 16,743 | -0.0028 (0.0034) | 0.397 |
| Winsorized | 16,956 | -0.0039 (0.0032) | 0.220 |
| **Glycine** |  |  |  |
| Primary | 16,956 | -0.0486 (0.0171) | 0.004 |
| Trimmed | 16,092 | -0.0447 (0.0223) | 0.046 |
| Winsorized | 16,956 | -0.0570 (0.0190) | 0.003 |

**Supplementary Table 3. Relationships of circulating choline, related metabolites, and dietary intake of choline with bone mineral density in the plasma vitamin D missing-indicator sensitivity analysis**

| Exposure | N | Coefficient (SE) | P value |
| --- | --- | --- | --- |
| Choline | 17,705 | -0.0041(0.0028) | 0.138 |
| Phosphatidylcholines | 17,705 | -0.0021(0.003) | 0.487 |
| Glycine | 17,705 | -0.0489(0.0167) | 0.003 |
| Dietary intake of choline | 17,705 | -0.0156(0.0102) | 0.128 |

**Supplementary Figures**

**Supplementary Figure 1. Mendelian randomization results for the association between the BMD and Choline metabolites in men and women.**


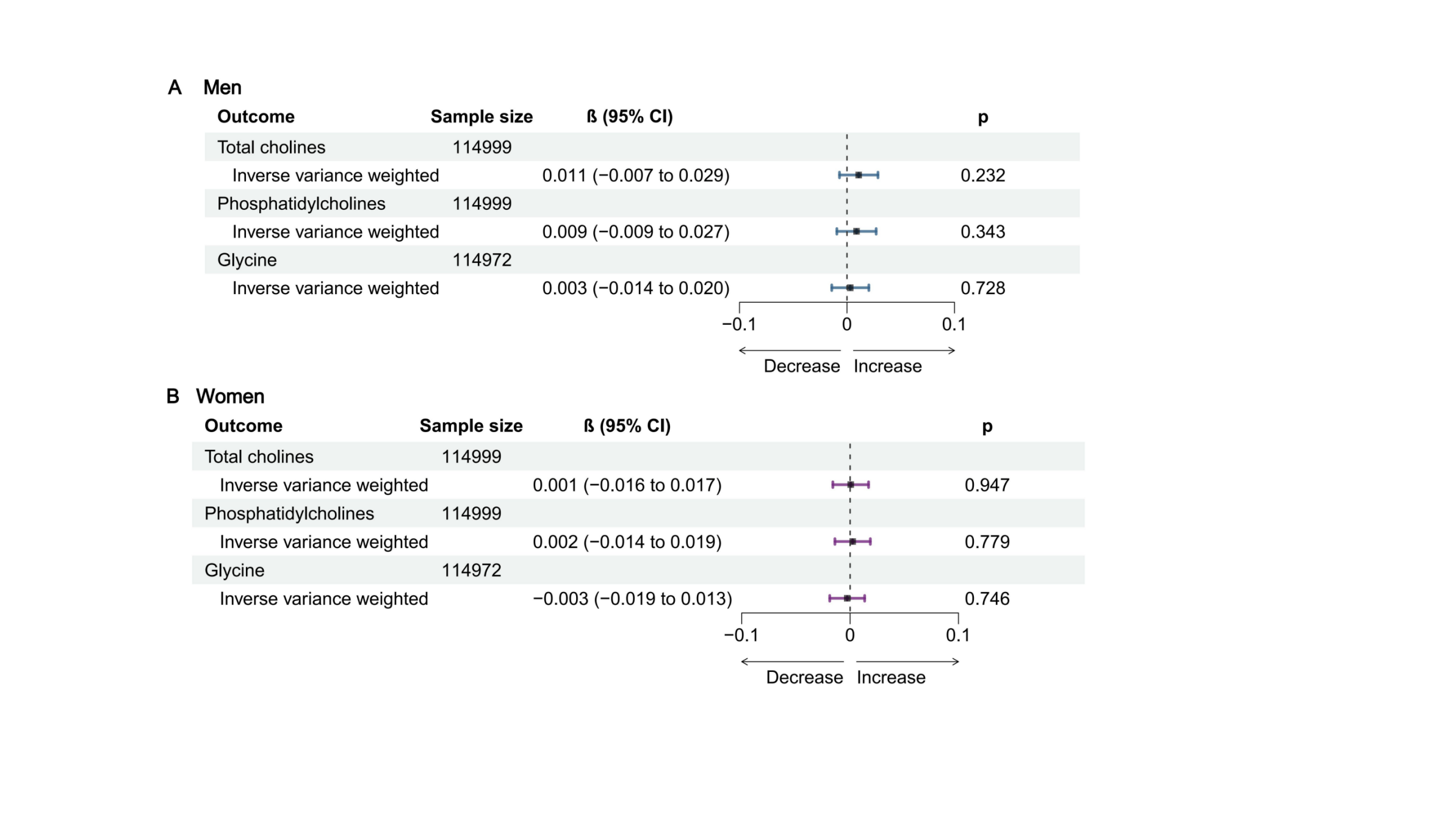


Abbreviations:BMD,bone mineral density; CI, confidence interval.
